# Environmental and Nutritional “Stressors” and Oligodendrocyte Dysfunction: Role of Mitochondrial and Endoplasmatic Reticulum Impairment

**DOI:** 10.3390/biomedicines8120553

**Published:** 2020-11-30

**Authors:** Jessica Maiuolo, Micaela Gliozzi, Vincenzo Musolino, Cristina Carresi, Saverio Nucera, Miriam Scicchitano, Federica Scarano, Francesca Bosco, Francesca Oppedisano, Roberta Macrì, Vincenzo Mollace

**Affiliations:** 1 Department of Health Sciences, Institute of Research for Food Safety & Health (IRC-FSH), University “Magna Græcia” of Catanzaro, Campus Universitario di Germaneto, 88100 Catanzaro, Italy; jessicamaiuolo@virgilio.it (J.M.); micaela.gliozzi@gmail.com (M.G.); xabaras3@hotmail.com (V.M.); carresi@unicz.it (C.C.); saverio.nucera@hotmail.it (S.N.); miriam.scicchitano@hotmail.it (M.S.); federicascar87@gmail.com (F.S.); francescabosco@libero.it (F.B.); oppedisanof@libero.it (F.O.); robertamacri85@gmail.com (R.M.); 2 Nutramed S.c.a.r.l, Complesso Ninì Barbieri, Roccelletta di Borgia, 88021 Catanzaro, Italy; 3 IRCCS San Raffaele, Via di Valcannuta 247, 00133 Rome, Italy

**Keywords:** oligodendrocytes, myelination, oxygen reactive species, endoplasmic reticulum, unfolded protein response, heavy metals, alcohol

## Abstract

Oligodendrocytes are myelinating cells of the central nervous system which are generated by progenitor oligodendrocytes as a result of maturation processes. The main function of mature oligodendrocytes is to produce myelin, a lipid-rich multi-lamellar membrane that wraps tightly around neuronal axons, insulating them and facilitating nerve conduction through saltatory propagation. The myelination process requires the consumption a large amount of energy and a high metabolic turnover. Mitochondria are essential organelles which regulate many cellular functions, including energy production through oxidative phosphorylation. Any mitochondrial dysfunction impacts cellular metabolism and negatively affects the health of the organism. If the functioning of the mitochondria is unbalanced, the myelination process is impaired. When myelination has finished, oligodendrocyte will have synthesized about 40% of the total lipids present in the brain. Since lipid synthesis occurs in the cellular endoplasmic reticulum, the dysfunction of this organelle can lead to partial or deficient myelination, triggering numerous neurodegenerative diseases. In this review, the induced malfunction of oligodendrocytes by harmful exogenous stimuli has been outlined. In particular, the effects of alcohol consumption and heavy metal intake are discussed. Furthermore, the response of the oligodendrocyte to excessive mitochondrial oxidative stress and to the altered regulation of the functioning of the endoplasmic reticulum will be explored.

## 1. Introduction

Oligodendrocytes are one of the main types of glial cells in the Central Nervous System (CNS) in addition to microglia and astroglia. Oligodendrocytes are the myelinating cells of the CNS and are derived from progenitor cells following extremely regulated processes of proliferation, differentiation, and migration. Oligodendrocytes have important functions in brain development by dealing not only with producing myelin but also with covering neuronal axonsm with the myelin itself electrically insulating the axons and consequently allowing for a rapid and easy transmission of the nerve impulse. In addition, oligodendrocytes offer essential trophic and metabolic support to neurons, with which they come into close contact [1,2,3]. Oligodendrocytes and astrocytes are perfectly integrated in the neural network, where they deal with the transport of the main ions, the release of many neurotransmitters, and the energy maintenance of neurons through the synthesis of glycogen. That way they can control the stability of the blood brain barrier (BBB) [4,5] and remove reactive oxygen species (ROS) [6,7]. During neural development, oligodendrocytes are initially present in progenitor forms (OPC) and appear, structurally, like bipolar cells. Newly formed OPCs, during their development, are characterized by the expression of some specific markers that will also characterize the future mature oligodendrocytes (OLs) [8]. A typical marker of OPCs is Platelet-Derived Growth Factor Receptor α (PDGFRα), a receptor located on the surface of many cell types, including mainly OPCs, but also intestinal, epithelial, and pulmonary cells [9]. This receptor binds to some Platelet-Derived Growth Factor isoforms, activating and acting as a mitogen and survival factor in OPCs, which, in this way, increases their number [8]. As development continues, OPCs transform into pre-oligodendrocytes, which interact with a target neuronal axon, lose their immobile bipolarity, and begin to build filamentous myelin protrusions. The pre-oligodendrocytes show two important markers arranged on the cell surface: O4 and O1, which are O4-O1 antigens positive. While O4 appears in a late phase of OPCs, O1 is characteristic of a pre-oligodendrocyte stage [10]. The next step involves the complete maturation of the pre-oligodendrocytes into mature oligodendrocytes, which changes their morphology from bipolar cells to highly branched cells, and produces myelin and associated proteins. This development phase is easily identifiable because mature cells express specific markers. Recent studies have identified genes for some transcription factors present only in mature oligodendrocytes, which regulate OL differentiation. To date, the best known are *UHRF1*, *ZFP536*, and *CDKN1C* [11].

Although mature oligodendrocytes have long been thought to be a homogeneous class of cells, there is currently a lot of information about their heterogeneity. In particular, three types of oligodendrocytes are known which reside in different locations and perform specific functions [3]:

- Interfascicular oligodendrocytes, which myelinate neuronal axons in white matter tracts;
- Perivascular oligodendrocytes which, which metabolically support axons;
- Perineuronal oligodendrocytes, which constitute a cellular pool for remyelination processes, if necessary, and regulate neuronal excitability.

Myelin, produced by mature oligodendrocytes, is a predominantly lipid membrane that wraps itself tightly around axons and induces their electrical isolation, promoting a jumpy propagation of the nerve impulse in points not covered by myelin, called Ranvier Nodes. In this way, impulse propagation is efficient and faster than that which occurs in unmyelinated axons [12]. The counterpart of oligodendrocytes, at the level of the Peripheral Nervous System (PNS), is constituted by Schwann cells, which provide isolation and neuronal metabolic support but, unlike oligodendrocytes, can myelinate one neuronal axon at a time [13].

The lipid content of myelin is very high and differs from all other eukaryotic plasma membranes; in particular, the lipid content constitutes about 70–75% of the dry weight of myelin, and lipids, cholesterol, phospholipids, galactolipids (galactolipid), and plasmalogen are present in the ratio 2:2:1:1 [14]. Reduced myelin lipid levels have been associated with axonal and oligodendrocyte degeneration and, consequently, with a reduction in white matter [15]. The major proteins present in myelin include Myelin Basic Protein (MBP, 30%), Proteolipid Protein 1 (PLP, 50%), Myelin Associated Glycoprotein (MAG), and Myelin-Oligodendrocyte Glycoprotein (MOG) [16]. All the mentioned proteins are essential to compact myelin and make it functional: the localization of these macromolecules moves on the plasma membranes during the development process that goes from pre-oligodendrocytes to mature oligodendrocytes. This shift is also enabled by an intact and functional cell cytoskeleton [17]. The subsequent adhesion between the oligodendrocyte and the neuronal axon is mediated by MBP and PLP myelin proteins. The proteins, therefore, are not static and inert molecules because, in addition to mediating the interactions between the intracellular sheets of myelin, they are their proteins and lipids for guiding the assembly of myelin. The loss of structural proteins has shown an altered stability of myelin up to the onset of demyelinating diseases, such as multiple sclerosis, Pelizaeus–Merzbacher Disease, Vanishing White Matter Disease, and Charcot–Marie–Tooth disease [18]. Like all cellular components, myelin undergoes turnover, and the proteins and lipids which compose it are replaced by newly formed molecules. The replacement of myelin has been defined as “myelin plasticity” [19]. The process of myelination is finely regulated and, from many studies reported in the scientific literature, it would appear that the mechanistic Target Of Rapamycin (mTOR) coordinates the synthesis of myelin proteins and lipids [20]. In particular, mTOR and its components (mTORC1 and mTORC2) promote the synthesis of lipids through the processing and maturation of the transcription factors Sterol Regulatory Element-Binding Proteins (SREBPs), which are normally responsible for the formation of lipids. When SREBPs are mature, therefore, they induce the expansion of numerous enzymes involved in the synthesis of fatty acids and cholesterol. mTORC1 signaling is also involved in the production of MBP; in fact, its alteration reduces the levels of MBP RNA, highlighting that the translation of this protein, and probably that of other myelinated proteins, is induced by mTORC1 [21]. Studies have shown that the loss of mature oligodendrocytes or their structural and functional alteration can easily lead to incorrect myelination and a predisposition to develop neurodegenerative diseases. The oligodendrocytic loss of the ability to migrate and communicate with other types of cells also leads to the same result [22]. Mitochondria are basically involved in energy support and the endoplasmic reticulum is the site of lipid synthesis, with both being fundamental for the maintenance of oligodendrocyte-neuron functionality [23,24].

## 2. Mitochondria and Endoplasmic Reticulum in Oligodendrocytes

Mitochondria are cellular organelles formed structurally by a double membrane and a mitochondrial matrix. Mammalian mitochondria contain over 1500 proteins, which vary in a tissue-dependent manner. These organelles also contain their own circular genome, mitochondrial DNA, which only encodes for 13 proteins. Thus, the mitochondria depend on the nucleus and other cell compartments for the production of their proteins and lipids. From a functional point of view, the main role of mitochondria is to generate energy for the cell. Mitochondrial dysfunction is associated with an increasingly larger proportion of diseases for humans, such as neurodegenerative disorders, cardiomyopathies, metabolic syndrome, cancer, and obesity [25].

In order to function at its best and perform all its functions properly the intricate neuronal network requires a significant amount of energy necessary to ensure the absorption of neurotransmitters, the maintenance of ion gradients, the generation of action potentials, the transport of organelles, the recycling of synaptic vesicles, and axonal growth. For this reason, the brain consumes 20–25% of our daily energy budget [26]. The white matter of the brain comprises about half of its volume and this is characterized by myelin-coated tissue. It is therefore evident how important the production of energy at the level of oligodendrocytes is. Mitochondria are essential organelles which regulate many cellular functions through oxidative phosphorylation. Mitochondria have been found to be fundamental in every moment of the development of oligodendrocytes:

- during the formation of myelin, they provide the substrates to generate the necessary and sufficient energy for the synthesis of lipids;
- during the development of brain functions, the mitochondrial production of ATP will be used to contribute to the support of axonal function;
- in the case of pathological changes, the mitochondria are able to buffer excess calcium ion or to induce the activation of apoptosis [27].

Neuronal axons can extend several feet in length and, for this reason, may require higher amounts of energy than is supplied by oligodendrocytes [23]. One hypothesis suggests that oligodendrocytes can transport glucose and pyruvate to neuronal axons to allow subsequent oxidative phosphorylation. The veracity of this theory has recently been demonstrated following experiments using coronal slices of mouse corpus callosum in a glucose deprivation model. These experiments highlighted that oligodendrocytes provide neuronal metabolic support under conditions of energy stress by transferring glucose via gap junctions [28]. Furthermore, another form of energy support could be the release of exosomes containing proteins, many of which are involved in energy metabolism [29]. Nonetheless, further evidence would need to be collected to determine whether oligodendrocyte glucose is subsequently metabolized by neurons. Recent scientific studies have described the characteristics of mitochondria in oligodendrocytes in the brains of rodents. In particular, oligodendrocytic mitochondria are found in narrow channels of the myelin sheath and are endowed with movement. However, the mitochondria of oligodendrocytes appear smaller, less abundant and with less mobility than the mitochondria found in neurons and astrocytes. These data report that the production of ATP in oligodendrocytes can contribute energetically to the formation of myelin supporting lipid metabolism [30]. Any mitochondrial dysfunction impacts cellular metabolism and negatively affects the health of the organism. In particular, mitochondrial dysfunction at the level of neurons causes oxidative damage responsible for cell death and, consequently, the onset of neurodegeneration [31]. In these cases, supplementation with antioxidant compounds of an exogenous or endogenous nature has been shown to reduce the severity of many neurodegenerative diseases [32]. What happens when mitochondrial dysfunction occurs in the glia? Today it is known that if the functioning of the mitochondria is unbalanced the myelination process is impaired; the main consequences are oligodendrocyte degeneration and the onset of CNS pathologies, such as ischemia and neurodegeneration [33]. Glutamate-induced excitoxicity, which precedes oxidative stress, caspase-3 activation and apoptotic death, is well known in neurodegenerative conditions [34,35,36]. Oligodendrocytes are also sensitive to glutamate excitotoxicity since these cells express the ionotropic α-amino-3-hydrox+y-5-methyl-4-isoxazolepropionic acid (AMPA)/kainite receptors, which are responsible for the overproduction of calcium ions, activation of the intrinsic mitochondrial pathway Bax- caspase 3 and apoptotic cell death [37].

At the end of the myelination process, oligodendrocytes synthesized about 40% of the total lipids present in the brain. The endoplasmic reticulum (ER) is a highly dynamic cellular organelle, consisting of tubules, sheets, and the nuclear membrane. It plays many roles in the cell including the storage of calcium, protein synthesis and lipids metabolism. For this reason, the alteration of this organelle can lead to partial or deficient myelination, triggering numerous neurodegenerative diseases. Several myelin proteins in the CNS and PNS are synthesized on ER-linked ribosomes, modified following translation and sent to myelin through vesicular trafficking [38]. The consequence is that the production of lipids and proteins, during myelination, greatly increases the load of the secretory pathway which is very susceptible to ER stress [39]. The altered production of myelin by oligodendrocytes leads, as already mentioned, to the death of these cells and the onset of demyelinating diseases. However, it is important to highlight that these diseases can also be generated as a result of a reduced ability of OPCs to differentiate into mature oligodendrocytes or altered processes of remyelination following injury or stressful conditions [40,41]. In addition, in many demyelinating diseases a correlation with ER stress has been demonstrated [38]. ER stress is caused by the accumulation of unfolded or misfolded proteins, by the alteration of lipid synthesis, by the loss of calcium deposits; in these conditions the cell responds with the activation of a pathway called Unfolded Protein Response (UPR) which heals ER stress by decreasing the protein load, the increased protein folding capacity and the induction of cytoprotective genes [42]. UPR protects the cell by reducing or eliminating ER stress. If UPR is unable to resolve the stressful events, the cell undergoes apoptosis [43]. For example, stressed ER is visible in oligodendrocytes of patients with multiple sclerosis, an autoimmune neurodegenerative disease that involves the formation of auto-antibodies to myelin is responsible for the gradual increase in disability. In fact, UPR activation has been found in the oligodendrocytes of these patients, as demonstrated by the involvement of transmembrane transducers of UPR [Protein kinase RNA-like Endoplasmic Reticulum Kinase (PERK), Inositol-Requiring kinase 1 (Ire1α), and Activating Transcription Factor 6 (ATF6)] [44]. Pelizaeus-Merzbacher disease (PMD) is a pathology linked to the X chromosome that causes the demyelination of the CNS due to a mutation in the gene that codes for the myelin protein PLP. The severity of this pathology depends on both the severity of the mutation and the ability of PLP to escape from the ER [45]. There are also PMD-like forms, in which mutations of genes that code for other myelin proteins, such as MAG. Additionally in this case there is the involvement of ER with an increase in UPR [46]. Charcot–Marie–Tooth disease encompasses a group of genetic pathologies caused by a mutation for a gene of peripheral myelin proteins; mutations can be expressed by Schwann myelinating cells in PNS [47] As in the case of PMD, the severity of these pathologies depends on how much the proteins are retained in the ER. Furthermore, there is a marked activation of UPR in Schwann cells [48]. To date, UPR can be considered as a therapeutic target for numerous neurodegenerative diseases that include myelin disorders [49]. These new therapies focus on the use of compounds that selectively modulate UPR transducers [50].

Each cellular organelle is in contact with the other organelles by means of specific proteins that connect them physically or actively participate in the communication of their functions. When contact is reached, organelles perform their functions to the best of their ability [51,52]. The role of ER-mitochondria contacts have been implicated in the interorganelle exchange of Ca^2+^, lipids, and various metabolites [53]. In the last fifty years, the ER-mitochondria contact responsible for the lipid transfer from one organelle to another has been defined and studied [54]. There are numerous proteins, that mediate ER-mitochondria contact, that have binding sites for lipids and transport domains. These proteins contain a synaptotagmin-like mitochondrial-lipid binding protein (SMP) domain and it has been shown that, among these, Mmm1 and Mdm12 bind phospholipids in vitro [55,56]. In this way, therefore, the described proteins serve to bind and facilitate the transfer of phospholipids between two membranes of organelles; in fact, it has been shown that cells without these components show defects in the transport of phospholipids between ER and mitochondria [57]. This concept is very important and could be an indispensable element in glio- and neurogenesis [58].

## 3. Environmental and Nutritional “Stressors” and Oligodendrocytes Impairment

Mature oligodendrocytes are sensitive to many factors such as the oxygen and iron concentrations, the excitotoxicity inductors, the ROS accumulation and unfolded/misfolded proteins. Consequently altered conditions may lead to metabolic disorders of oligodendrocytes up to irreversible damage [59]. From this point of view, habits and lifestyle are essential to maintain a condition of oligodendrocyte balance.

Nutritional habits, for example, greatly influence the development of the neuronal network and may have effects before neurological disorders become clinically evident: for this reason, it is difficult to determine a cause-effect relationship. Western countries’ diet is hypercaloric and rich in saturated fat, carbohydrates and processed foods [60]. In particular, several studies related the diet to multiple sclerosis and it was highlighted that patients with this disease consumed less fruit, whole grains and vegetables compared to nutrition guide claims; in addition, a reduced fat consumption led to lower levels of disease [61].

Smoking is also considered a risk factor for neurogenesis and myelination [62]. In a clinical study of 350 individuals, the smoking status was assessed in relation to the number of cigarettes smoked daily and the number of years in which this habit is present. The results showed that 30% of patients suffering from demyelinating diseases were habitual smokers [63].

Physical inactivity is defined as the decrease in body movement that produces a decrease in energy expenditure and may causes risk factors that increase morbidity and mortality [64]. Beyboun et al. demonstrated that a reduction in physical activity is a risk factor for Alzheimer’s disease and that 31.9% of these individuals developed dementia [65]. In addition, a clinical study found that, in individuals with overt depression, exercise resulted in reduced symptoms [66].

Finally, in a clinical study conducted on 8983 participants with demyelinating diseases, 17.3% were smokers 18.2% were abusing chemicals and only 25% had regular physical activity. The harmful impact of bad habits may be related to (1) reduced antioxidant activity and (2) increased inflammatory processes [67].

In the next section, we will deepen two aspects in which oligodendrocytes and/or OPCs can suffer: chronic alcohol consumption and heavy metal intake.

### 3.1. Chronic Consumption of Alcohol and Oligodendrocytes

It has been widely demonstrated that chronic alcohol intake constitutes a huge public health problem all over the world and is a major cause of the onset of diseases and premature death. The effects of alcohol can vary greatly in relation to the daily amount and type of consumed drink. To date, the adverse effects of alcohol consumption on various parts of the body are known including the cardiovascular system, immune system, liver, intestine, and nervous system [67,68,69,70,71,72,73]. In particular, alcohol is regarded as a negative CNS modulator, since it has been shown that prolonged alcohol consumption can induce neuronal death and impaired brain development [74]. Among CNS cells, astrocytes, oligodendrocytes and synaptic terminals are the main targets of the alcohol and alterations are represented by white matter atrophy, neural inflammation and toxicity, and synaptogenesis alterations [75]. Scientific tests conducted in recent years have shown that alcohol consumption was related to the reduction in white matter and to an incorrect myelin composition and myelin functionality [76]. Obtained findings from post-mortem studies of alcoholics confirmed a reduction in brain weight and increased demyelination [77]. Furthermore, alterations of the myelin structure with a reduced presence of sphingolipids and phospholipids, an increased presence of vacuoles, and mitochondrial alterations were highlighted [78]. There is much scientific relevance on the basis of which the consumption of large quantities of alcohol determines myelin changes also in the fetus [79]. In fact, in vivo studies have shown, in a mouse model exposed to alcoholic quantities corresponding to those of an alcoholic subject, that the fetuses in the third trimester showed a 58% reduction in mature oligodendrocytes and 75% of OPCs. Moreover, studies conducted in vitro on OPCs have shown that treatment with EtOH negatively modulated the expression of PDGFRα, which has a fundamental role in the differentiation of OPC in mature and myelinating oligodendrocytes [80]. Scientific evidence has shown that oxidative stress, ER stress and apoptotic-caspase3-dependent death were increased in the brain in a model of mice which were given free access to ethanol and water (in the ratio 1:9, respectively) for seven months [81]. Finally, it is important to report that a prolonged abstinence from alcohol was able to positively modify the structure of myelin: in this case, it was possible to observe a new increase in the expression of the MBP myelin protein in rats [82].

Chronic excessive consumption of alcohol causes injury to many tissues, including the liver, brain, heart, or lung. There is considerable evidence, from both human and animal studies, that alcohol consumption enhances oxidative stress and alters mitochondrial function. In fact, chronic alcohol consumption limits mitochondrial oxidative phosphorylation by suppressing the translation of proteic complexes such as NADH dehydrogenase (Complex I), cytochrome b-c1 (Complex III) cytochrome oxidase (Complex IV) or the ATP synthase complex (Complex V) [83]. Excessive alcohol consumption involves alterations also in the endoplasmic reticulum. The stress of this organelle has been implicated in various central nervous system lesions, including cerebral ischemia, traumatic brain lesions, and neurodegenerative and neurovascular disorders associated with aging [84]. A stress condition of the endoplasmic reticulum, induced by the consumption of alcohol, could involve an increase in the number of unfolded or misfolded proteins, an alteration in calcium deposits, and a reduction in lipid metabolism [85].

### 3.2. Intake of Heavy Metals and Oligodendrocytes

Heavy metals are normally present in the Earth’s crust, and they are released spontaneously in concentrations that do not affect human health. However, any metal can be considered a “contaminant” if its concentration causes a harmful effect on humans or the environment [86]. To date, the presence of heavy metals in the environment has increased exponentially as a result of human activity, which includes industrialization, anthropogenic waste, and the use of fertilizers and pesticides. It is also worth mentioning the occupational exposure to heavy metals that occurs in the workplace [87]. The main metals considered dangerous if present in abundant concentrations are lead (Pb), cadmium (Cd), mercury (Hg), arsenic (As), chromium (Cr), copper (Cu), nickel (Ni), aluminum (Al), cesium (Cs), cobalt (Co), molybdenum (Mo), strontium (Sr), and uranium (U) [88].

**Figure 1:**
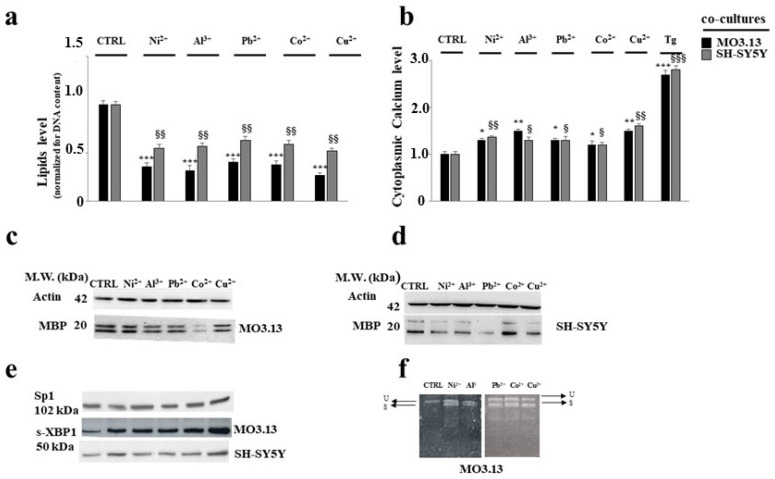
Alterations induced by heavy metals on oligodendrocytes and neurons grown in co-culture. In particular, panel (**a**) shows the reduction in lipid content compared to the control. In panel (**b**), an increase in the basal concentration of the calcium ion is shown. * denotes *p* < 0.05 vs. control; ** denotes *p* < 0.01 vs. control; *** denotes *p* < 0.001 vs. control. § denotes *p* < 0.05 vs. control (grey bars); §§ denotes *p* < 0.01 vs. control (grey bars); §§§ denotes *p* < 0.001 vs. control (grey bars). Panels (**c**,**d**) highlight the reduction in myelin MBP protein expression. In panels (**e**,**f**), the involvement of s-XBP1 expression were evaluated. The presence of possible XBP1 mRNA splicing by RT-PCR was also highlighted (panel f). The modulations observed in the picture were statistically significant. Values of three independent experiments are expressed as mean ± standard deviation (sd). The figure is taken and modified from [24,95].

Heavy metals are very resistant to biological and chemical decomposition processes due to their non-degradable nature; the consequence is their accumulation in the food chain through the process of bio-magnification [89]. Heavy metals can accumulate in various organs, including the liver, heart, kidney, and brain, and greatly affect their function. In the last few decades, the presence of neurological damage following acute and chronic exposure to heavy metals has been increasingly evident [90,91]. In particular, it has been shown that the accumulation of some metals can alter the process of myelination, possibly leading to neurodegeneration [92,93]. An important scientific work conducted in our research group and recently published has been carried out in vitro on mature human oligodendrocytes and human neurons grown individually and in co-culture; cell lines were exposed to non-toxic concentrations of several essential (Cu^2+^, Cr^3 +^, Ni^2+^, Co^2+^) and non-essential (Pb^2+^, Cd^2+^, Al^3+^) heavy metals. The choice to use sub-toxic concentrations of heavy metals was motivated by the desire to reproduce a model in which cells were in contact with the concentrations to which humans are exposed daily and unconsciously [94]. The results of this study showed that oligodendrocytes are more susceptible than neurons to exposure to heavy metals when the two cell lines have grown in direct contact. In addition, exposure to heavy metals showed a reduced oligodendrocyte expression of the MBP myelin protein and a massive dysregulation of the calcium ion in the ER. With this scientific study, the interruption of the cross-talk between oligodendrocytes and neurons has been demonstrated when cells are exposed to some heavy metals. The continuation of this work wanted to investigate whether UPR was involved. IRE1α was found to be activated and our results have shown that this arm of the UPR carries out a protective action against the insult generated by the treatment with heavy metals. In fact, the silencing of IRE1α determined an increased mortality in both neurons and oligodendrocytes compared to the mortality observed in the absence of silencing. Furthermore a consequence of the silencing of IRE1α was the increase in ROS and index of lipid peroxidation not observed in the absence of silencing. Finally, the administration of subtoxic concentrations of heavy metals demonstrated the involvement of SREBP1, a transcription factor able to upregulate the synthesis of enzymes involved in phospholipid biosynthesis. SREBP1 was found to be active in both neurons and oligodendrocytes [24]. Figure 1, taken from papers already published by our group, show damage following heavy metal treatment in oligodendrocytes and neurons and the involvement of the endoplasmic reticulum [24,95].

It has been shown that heavy metals may be responsible for the induction of oxidative stress, with the production and accumulation of ROS, increased cytotoxicity, and genotoxic stress that involves physical or chemical alterations to the DNA [96]. Furthermore, experiments conducted on fetuses and embryos have shown that exposure for a short time to cadmium (25–100 μM) alters the stability of oligodendrocytes and induces apoptotic-mitochondria-dependent death in OPCs. In this cell line, nuclear condensation, DNA fragmentation, the mitochondrial release of cytochrome c, and the activation of caspase 3 and 9 were evaluated. Cadmium, therefore, is responsible for altered myelination resulting from mitochondrial suffering probably due to oxidative stress [97]. Mercury also acts negatively on the correct functioning of the brain: the best-known model of multiple sclerosis (autoimmune encephalomyelitis, EAE) was experimentally induced in groups of mice. The obtained results showed high mitochondrial toxicity with the accumulation of ROS, morphological alterations, the release of cytochrome c, and cell apoptotic death. In this case, therefore, repeated exposure to mercury accelerated the progression of multiple sclerosis through oxidative damage [98]. Lead is a well-known heavy metal for its neuro-toxic properties. In fact, high intakes of this metal cause cognitive, neuro-behavioral, and motor disorders dependent on the absorbed and accumulated quantities. These deleterious effects are particularly present if lead is accumulated during childhood or in the age of development [99]. The tolerated levels of lead in the blood of adults are 70 μg/dL and higher values are the cause of encephalopathy. In these cases, magnetic resonance imaging (MRI) can detect macroscopic brain abnormalities [100]. Numerous experimental studies conducted in vitro and in vivo have highlighted the toxicity of lead both in neurons and in glia. In mature oligodendrocytes, lead induces the delayed differentiation of OPCs and promotes an altered structure of mature oligodendrocytes. Finally, the disintegration of the multi-lamellar structure was found after exposure to lead [101].

**Figure 2:**
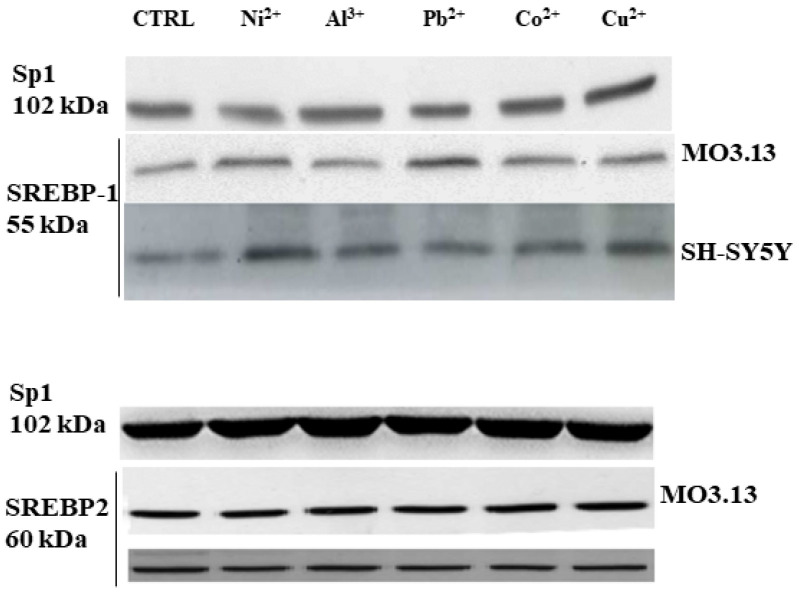
Involvement of SREBP-1 transcription factor induced by heavy metals on oligodendrocytes and neurons grown in co-culture. The panel shows the involvement of SREBP-1 but not of SREBP-2 in this experimental system. The figure is taken and modified from [24].

Figure 2, taken from paper already published by our group, shows the involvement of the transcription factor SREBP-1 but not of SREBP-2 in the damage generated by the treatment of heavy metals in oligodendrocytes and neurons [24].

The functional alteration of mature oligodendrocytes and OPCs is known following the massive uptake of all heavy metals, although a greater amount of data should be produced and the mechanisms of action and possible interactions with DNA should be better explored.

## 4. Discussion and Conclusions

In this review, the fundamental role played by oligodendrocytes in all phases of life was outlined. The integrity of these cells guarantees the development and correct functioning of the CNS [102,103,104]. Embryological studies have confirmed that oligodendrocytes, in order to become mature and functional, need to evolve from OPCs, and these maturation steps are extremely and finely regulated. Although this argument is continuously updated, many doubts still exist, and it would seem that there is no single mechanism to regulate the maturation process of oligodendrocytes. Once this development is completed, axonal myelination by oligodendrocytes takes place and this process must also be finely regulated in order to avoid demyelinating diseases and neurodegeneration. In this delicate period of time, any oligodendrocyte dysfunction can be responsible for structural and functional alterations [105,106]. Numerous scientific studies have shown how a correct lifestyle is essential to protect the good health of oligodendrocytes. This habit should be followed by every adult person in order to maintain and not alter the oligodendrocyte balance already formed [107,108]. Furthermore, an adequate lifestyle should also be followed by pregnant women, since this period coincides with the initial formation and development of the nervous system of the fetus [109,110]. Finally, it is also important that correct habits are maintained during childhood, since this age group is important for the continuation of a correct development of the system nervous [111]. Incorrect habits and unaware daily exposures to alcohol and heavy metals, respectively, can lead to damage to oligodendrocytes. These two aspects have been discussed in the current review, but there are other deleterious factors, such as smoking, excessive exposure to environmental pollution, and the use of drugs. In particular, tobacco smoke increases the extent of inflammatory processes, participating in the destruction of the main myelin proteins, mainly including MBP. It has also been shown that smoking worsens the course of demyelinating diseases, such as multiple sclerosis [112,113,114]. Even exposure to pollution has been shown to have deleterious effects on oligodendrocytes and myelination [115,116]. Finally, drug abuse with psychotic effects on the central nervous system can interact the oligodendrocytes, even if this aspect remains controversial [117,118].

In this review we have highlighted how the main damages and malfunctions of oligodendrocytes are related to the dysfunction of two fundamental cellular organelles: the mitochondrion and the endoplasmic reticulum.

In light of what has been deepened and described, there are two directions in which research in this area could continue:

(1) Beginning to consider mitochondria and the endoplasmic reticulum as the main targets of oligodendrocyte dysfunction and trying to restore their conditions in order to evaluate any positive modulations in various neurological pathologies;
(2) Changing the direction in which neurological pathologies are observed—from the oligodendrocyte to the neuron, instead of from the neuron to the oligodendrocyte. In this way, oligodendrocytes, astrocytes, and microglia could be considered as the reference point of the neuron in health and disease conditions.
